# Neonatal mortality rates, characteristics, and risk factors for neonatal deaths in Ghana: analyses of data from two health and demographic surveillance systems

**DOI:** 10.1080/16549716.2021.1938871

**Published:** 2021-07-26

**Authors:** Shadrach Dare, Abraham R. Oduro, Seth Owusu-Agyei, Daniel F. Mackay, Laurence Gruer, Alfred Kwesi Manyeh, Ernest Nettey, James F. Phillips, Kwaku Poku Asante, Paul Welaga, Jill P. Pell

**Affiliations:** a Mother and Infant Research Unit, School of Health Sciences, University of Dundee, Dundee, UK; b Navrongo Health Research Centre, Ghana Health Service, Navrongo, Ghana; c Kintampo Health Research Centre, Ghana Health Service, Kintampo, Ghana; d Institute of Health Research, University of Health and Allied Sciences, Ho, Ghana; e Institute of Health and Wellbeing, University of Glasgow, Glasgow, UK; f Mailman School of Public Health, Columbia University, New York, NY, USA

**Keywords:** Neonatal mortality rate, cause of death, risk factors, Ghana, child health

## Abstract

**Background:**

Reducing neonatal mortality rates (NMR) in developing countries is a key global health goal, but weak registration systems in the region stifle public health efforts.

**Objective:**

To calculate NMRs, investigate modifiable risk factors, and explore neonatal deaths by place of birth and death, and cause of death in two administrative areas in Ghana.

**Methods:**

Data on livebirths were extracted from the health and demographic surveillance systems in Navrongo (2004–2012) and Kintampo (2005–2010). Cause of death was determined from neonatal verbal autopsy forms. Univariable and multivariable logistic regression were used to analyse factors associated with neonatal death. Multiple imputations were used to address missing data.

**Results:**

The overall NMR was 18.8 in Navrongo (17,016 live births, 320 deaths) and 12.5 in Kintampo (11,207 live births, 140 deaths). The annual NMR declined in both areas. 54.7% of the births occurred in health facilities. 70.9% of deaths occurred in the first week. The main causes of death were infection (NMR 4.3), asphyxia (NMR 3.7) and prematurity (NMR 2.2). The risk of death was higher among hospital births than home births: Navrongo (adjusted OR 1.14, 95% CI: 1.03–1.25, p = 0.01); Kintampo (adjusted OR 1.76, 95% CI: 1.55–2.00, p < 0.01). However, a majority of deaths occurred at home (Navrongo 61.3%; Kintampo 50.7%). Among hospital births dying in hospital, the leading cause of death was asphyxia; among hospital and home births dying at home, it was infection.

**Conclusion:**

The NMR in these two areas of Ghana reduced over time. Preventing deaths by asphyxia and infection should be prioritised, centred respectively on improving post-delivery care in health facilities and subsequent post-natal care at home.

## Background

The fourth Millennium Development Goal of reducing the global child mortality rate by two-thirds between 1990 and 2015 could not be achieved, in part because of the continuing high neonatal mortality rate (NMR) in developing countries. NMR is defined as the proportion of deaths within 28 days of delivery per 1,000 live births [1]. Nearly half (47%) of deaths in children under-five years of age occur within 28 days of birth and almost all (98%) of these occur in developing countries [1]. In Ghana, neonatal deaths account for nearly half of deaths among under-five year olds [2]. If the current trend persists, there will be 30 million neonatal deaths globally between 2017 and 2030 [1]. Therefore, reducing NMRs in developing countries remains a key global health goal. The United Nations Sustainable Development Goal (SDG) 3.2 aims to ‘end preventable deaths of newborns and children under five years of age’ by 2030, ‘with all countries aiming to reduce their NMR to at least as low as 12 per 1,000 live births’ [3]. The Government of Ghana has enacted the SDGs and is committed to reducing newborn deaths [4].

Neonatal infection, preterm birth, low birth weight, and birth asphyxia are known leading causes of neonatal deaths worldwide [1]. Asphyxia and prematurity are mainly responsible for neonatal deaths within the first week of life- the early neonatal period- when most neonatal deaths occur; infection is the major cause of death after seven days of birth- the late neonatal period. Studies have shown that maternal characteristics, including age and education [5], place of delivery [6] and type and time of initiation of breastfeeding [7] are associated with neonatal mortality. There is also evidence that the survival of female neonates may be better than males [8]. Since the risk factors and causes of neonatal mortality differ with availability, access and quality of health care, understanding neonatal mortality in relation to these factors in countries with high NMRs is crucial.

The lack of reliable data in developing countries is a major hindrance to accurate assessment of newborn deaths. Previous studies have also been riddled with methodological challenges that make it difficult to reach firm conclusions. For example, sub-Saharan Africa has the highest NMR in the world, yet some of the weakest registration systems for health and vital events [1,9]. Most newborn deaths occur at home and are unrecorded [10,11]. NMRs are thus often estimated by extrapolating from hospital-based studies or surveys that use cluster sampling techniques [12,13]. As a result, NMRs often tend to be underestimated. In addition, data collected in cross-sectional studies may suffer recall bias, be of poor quality or may not reflect the temporal ordering of risk factors. With less than ten years to end the SDGs, unless the characteristics of neonatal deaths are clearly understood and the factors contributing to these deaths established, planning appropriate community level interventions is extremely difficult. Determining the true burden of neonatal mortality, the causes of those deaths, and their characteristics using reliable community level data is, therefore, crucial to implementation and monitoring progress towards set goals.

Unlike many developing countries, Ghana has three health and demographic surveillance systems (HDSS) [14–16] located in Navrongo (northern Ghana), Kintampo (central Ghana) and Dodowa (southern Ghana). They collect information on key health and sociodemographic factors including a range of data on pregnancy and birth. Standardised neonatal verbal autopsy (VA) questionnaires are used to collect information about neonatal deaths that allow the most probable cause of death to be determined and coded [17]. With the availability of these datasets, we set out to carry out a detailed study of neonatal deaths in Ghana. This would include calculating NMRs, analysing their causes and circumstances, and identifying any modifiable associated factors with the aim of preventing future neonatal deaths.

## Methods

### Health and demographic surveillance systems

In each HDSS study area, unique ID numbers are assigned to every household and their respective members. Trained field workers visit all registered households regularly – every four to six months – to collect key sociodemographic and health data. In addition, key events between visits are reported by trained community key informants. Demographic data are compiled that update the population under observation: household membership as altered by births, deaths, and migration into or out of households. Ancillary data are routinely collected on marriage, socio-economic indicators and key health variables such as vaccination. In the case of a neonatal death, a special neonatal verbal autopsy questionnaire is completed by a field supervisor who interviews the immediate caregiver of the deceased or another eligible informant in the household. These verbal autopsy questionnaires are then independently reviewed by at least two doctors to assign cause of death. Where there is disagreement between the doctors on the cause of death, a third is consulted. Data quality in the HDSSs is assessed by the field supervisor who does on-site cross-checks of any queries from the data processing unit in the research centres. As a result of these measures, the HDSS are a credible source of determining vital events within their catchment areas. Detailed descriptions of the methods employed in the HDSS have been previously published [14–16].

Data on all livebirths were extracted from HDSS registers from Navrongo (2004–2012) and Kintampo HDSS (2005–2010). Unfortunately, the proportion of missing and unvalidated data for many variables in the Dodowa HDSS dataset covering the study period was too great for it to be used in this analysis. The Navrongo HDSS covers the East and West Kassena-Nankana districts in the Upper-East region of Northern Ghana. The surveillance area comprises two major ethnic groups- the Kassems in the east and the Nankans in the west. The area has largely savannah vegetation with short periods of rainfall and a prolonged dry season. About 80% of the area is rural with the local economy mainly comprising subsistence farming and tourist attraction sites. The area has one referral district hospital, seven health centres and 27 community health compounds. The road network in Navrongo is largely dirt feeder roads and tracks with asphalted roads between major towns and the main means of transport is motor bike. For this study, we obtained an electronic extract of the Navrongo HDSS dataset covering the Kassena-Nankana East district. The Navrongo HDSS ethics committee considered that information from the Kassena-Nankana East, which is the larger of the two districts, was sufficient to answer the study research questions.

The Kintampo HDSS covers Kintampo North and South districts in the Bono East region of Central Ghana. The area lies on the forest-savannah transitional ecological belt. The major tribe in the area is Akan. About a third of the KHDSS is urban and the remainder rural. There are two district hospitals in the Kintampo HDSS. The road network in Kintampo is largely dirt feeder roads and tracks with asphalted roads between major towns. The main means of transport is by car- including taxis. In this study, we obtained an electronic extract of the Kintampo dataset covering both Kintampo North and South districts. Box 1 summarises the definition of the variables recorded by each HDSS.

**Box 1.** Definition of variables

**Outcome variable**: ***Neonatal death***: Neonatal death is defined as the death of a baby within 28 days of life (days 0–27). In this study it was dichotomised as Dead [coded as 1] if the baby died within 28 days or Alive [coded as Not dead = 0] if (s)he did not die within the study period or died after 28 days of birth. Deaths within seven days of birth (days 0–6) were classified as early neonatal deaths (END) and deaths after seven days (days 7–27) were considered late neonatal deaths (LND). **Explanatory variables**: ***Place of delivery***: Refers to the setting where the baby was reported to have been delivered, categorised as hospital, clinic or home births. Hospitals were formal facilities with a midwife or nurse and a doctor. Clinics were formal facilities with either a midwife or nurse, but no doctor. It included health centres, community-based health planning services (CHPS) and maternity homes. Home births included babies delivered in either the traditional birth attendant’s (TBA) home or the mother’s home, with no formally trained midwife or doctor. ***Health facility birth***: A collective term for hospital and clinic births ***Place of birth/ place of death***: This was a derived variable based on where the baby was born and where (s)he died. For example, Hosp/Hosp referred to a baby who was born in a hospital and died in a hospital; Hosp/Home referred to a baby who was born in a hospital but died at home; Home/Hosp referred to a baby who was born at home but died in a hospital. ***Socioeconomic status (SES***): SES was derived by statisticians at the research centre from principal component analyses (PCA) of household assets: type of housing; roofing and flooring material; structural condition of house; number of rooms; status of occupancy; electricity supply; type of cooking fuel; household facilities; source of water; health insurance enrolment status; land ownership and utilisation; type of assets in working conditions; income per week or month of household head and other income earners in the household; type of cooking salt; livestock and number of leave days. In this study SES was classified as five wealth quintiles for the study population: Poor, Next poor, Average, Next rich and Rich. ***Marital status***: Marital status was dichotomised as married or not married. Data for marital status were only available in the Navrongo HDSS. ***Maternal age***: Mother’s age at delivery was calculated as the difference between baby’s year of birth and mother’s year of birth. Mother’s age recorded as younger than 13 years old or older than 60 years was considered likely to be incorrect and treated as missing (n = 24). ***Education***: Education referred to the highest level of formal education attained by the mother at the time of delivery. This was categorised as: No education: a mother who had received no form of schooling; Basic education: a mother who had attained up to junior high education; Secondary or more: a mother who had received secondary education or higher. This information was only available for Navrongo for the study period. ***Antenatal care* (ANC) *attendance***: ANC attendance was classified as Yes or No, depending on whether a mother received at least one episode of ANC during pregnancy. This was recorded after delivery, based on maternal recall. In Navrongo, it was not recorded before 2012. ***Multiple births***: Multiple births refer to a mother who delivered more than one baby at the end of a single pregnancy. It was dichotomised into singletons and multiple, for twins or more. If a multiple birth was recorded as more than four babies, it was judged to have been an error and treated as a missing value. ***First birth***: First birth was derived from the birth order of the baby. This was dichotomised as Yes or No, if the baby was the first born or not. ***Parity***: Parity refers to the total number of live births a woman had delivered, including the current birth, at the time of data collection. It was categorised into 1, 2, 3 and 4 or more, and the value was cross-checked with *first birth* for consistency (*Parity *= 1 if *first birth *= Yes; *Parity*>1 if *first birth *= No). If *parity* was greater than 1 and *first birth = *Yes, the value of *parity* was treated as missing. ***Sex***: Male or female. ***Day of birth***: Refers to the day of the week in which a baby was born. It was generated from the baby’s date of birth and categorised as weekend if a baby was born on Saturday or Sunday, and weekday if (s)he was born from Monday to Friday inclusive. ***Year of birth***: Refers to the year the baby was born. We assessed for linearity of year of birth in the univariate models and observed that it was held for Navrongo, but not Kintampo. Therefore, year of birth was modelled as a continuous variable in Navrongo, and as a dummy in Kintampo. **Age at death**: Age at death was calculated as the difference, in days, between date of birth and date of death. It was categorised into 0 (deaths on the day of birth), 1–6 (early neonatal deaths, excluding deaths on the day of birth) and 7–27 days (late neonatal deaths). Cause of death*: Refers to the most probable cause of neonatal death as ascertained by at least two medical doctors from the VA narrative and checklist. *All the variables, except cause of death, were reported by the parent, usually the mother.

Although the HDSS employ a common core software system, there were some differences in how the variables were measured or coded. Data for marital status and maternal education were only collected in Navrongo. The socioeconomic status (SES) of a mother was based on a principal component analysis of social class, household assets and utilities. Given the differences in resource availability, housing structure and economic activities across northern and central Ghana, items collected across the regions differed. In addition, SES data were supplied as quintiles for each HDSS area. It was thus possible that a woman classified as rich in Navrongo could be average in Kintampo. Conducting multivariable analyses on the combined data from the two centres would also mean that important variables would be omitted, or the sample size reduced when data were restricted to the period when data were available in both HDSS, which would yield biased results. Due to these differences, it was not feasible to combine the data from the two HDSS or to formally investigate inequalities between the two research sites. They were therefore analysed separately.

### Statistical analyses

The differences between maternal, delivery and neonatal characteristics and neonatal death was tested using Pearson’s chi square tests for categorical variables (place of delivery, sex, antenatal clinic visits, multiple birth, first birth, day of birth, marital status, and occupation) and chi square tests for trend for ordinal variables (education, maternal age, SES, parity, and year of birth). Bar graphs show the distribution of neonatal deaths according to age at death, place of birth or death, and causes of death.

Binary logistic regression models were used to explore the associations between maternal, delivery and neonatal characteristics, and neonatal mortality [coded as Dead = 1; Alive = 0]. Univariable models were supplemented with multivariable models to adjust for potentially confounding variables. The variables included in the multivariable models were dependent on what had been provided by each HDSS. The multivariable models used cluster robust standard errors to account for the possible effect of clustering at the level of place of delivery. Since neonatal mortality was a relatively rare outcome in the population (less than 10%), odds ratios are appropriate to estimate the risk of death [18,19], and ‘any qualitative judgment is unaltered by the discrepancy between the odds ratio and the relative risk’ [19].

Missing data were analysed using multiple imputation techniques [20] conducted using Stata’s imputations by chained equations (ICE). ICE algorithms were used because they do not assume a joint multivariate normal distribution but allow specification of the conditional distribution of each variable to be imputed. This was particularly useful because the explanatory variables being imputed in the current model included a combination of count data (parity), binary variables (sex), and ordinal variables (SES). The number of imputations (m = N) generated was based on the fraction of missing information (FMI) [21]. Fifty imputations were created in the Navrongo model, and 40 in the Kintampo model. The next phase of the multiple imputation process is to run the analytical model of interest on each of the imputed datasets to generate parameter estimates and standard errors. This analytical model runs on each imputed dataset as though they were independent complete datasets. The parameter estimates and the standard errors calculated from each imputed dataset and the original dataset were summed into a single estimate using Rubin’s rule. When we computed the Akaike Information Criterion (AIC) and Schwarz’ Bayesian Information Criterion (BIC) for the original and the imputed model, it was seen that the imputed model was preferred. Since the distribution of variables was likely to vary between HDSS areas, the HDSS data were imputed separately for each HDSS area. A Hosmer-Lameshow test was used to check the adequacy of the final models. Likelihood ratio tests for statistical interactions by maternal age, SES, parity and year of birth were conducted, and where statistically significant differences were found, appropriate subgroup analyses were conducted. All statistical analyses were conducted using Stata version 14SE (StataCorp, Texas, USA) and statistical significance was set at 5% (p < 0.05).

## Results

Table 1 summarises the characteristics of mothers and babies, neonatal deaths and distribution of overall NMRs in Navrongo (2004–12) and Kintampo (2005–10). Over the study periods, 28,410 live births were delivered: 17,153 in Navrongo and 11,257 in Kintampo. After excluding babies delivered on the way to hospital or clinic (n = 22), and those delivered in unspecified (n = 165) or unknown (n = 3) facilities, the study population comprised 28,223 live births: 17,016 in Navrongo and 11,207 in Kintampo. In Navrongo, (10,014 babies (58.9%) were born in a hospital or clinic and 7,002 (41.1%) at home; in Kintampo, 5,424 babies (48.4%) were born in a hospital or clinic and 5783 (51.6%) at home.

**Table 1.** Summary of maternal and neonatal characteristics, neonatal deaths, and overall NMRs in Navrongo (2004–2012) and Kintampo (2005–10)

	Navrongo				Kintampo			
	Total livebirths	Dead	NMR	p-value	Total livebirths	Dead	NMR	p-value
	n (%)	n (%)			n (%)	n (%)		
**Total**	17,016	320	18.8	-	11,207	140	12.5	-
**Place of birth**								
Home	7,002 (41.1)	139 (43.4)	19.9	5,783 (51.6)	58 (41.4)	10.0		
Hospital	7,306 (42.9)	150 (46.9)	20.5	0.01	3,634 (32.4)	64 (45.7)	17.6	0.003
Clinic	2,708 (15.9)	31 (9.7)	11.4	1,790 (16.0)	18 (12.9)	10.1		
**Maternal age**								
<20 years	1,875 (11.0)	52 (16.3)	27.7	0.07	963 (8.6)	8 (5.8)	8.3	<0.001
20–29	8,357 (44.1)	141 (44.1)	16.9	5,344 (47.9)	55 (39.6)	10.3		
30–39	5,229 (30.7)	106 (33.1)	20.3	3,994 (35.8)	54 (38.9)	13.5		
40+	1,555 (9.1)	21 (6.6)	13.5	863 (7.7)	22 (15.8)	25.5		
Missing	-	-	43	1				
**Socioeconomic status**								
Poor	3,855 (24.3)	61 (26.0)	15.8	0.34	1,811 (16.2)	24 (17.3)	13.3	0.40
Next poor	2,916 (18.3)	47 (20.0)	16.1	2,138 (19.1)	22 (15.8)	10.3		
Average	2,777 (17.5)	37 (15.7)	13.3	2,311 (20.7)	24 (17.3)	10.4		
Next rich	3,205 (20.2)	49 (20.9)	15.3	2,509 (22.5)	35 (25.2)	13.9		
Rich	3,146 (19.8)	41 (17.5)	13.0	2,406 (21.5)	34 (24.5)	14.1		
Missing	1,117	85	32	1				
**Marital status**								
Not married	2,736 (16.1)	65 (20.3)	23.8	0.04	-	-	-	
Married	14,280 (83.9)	255 (79.7)	17.9	-	-	-		
**Education**								
None	4,926 (29.6)	99 (31.6)	20.1	0.11	-	-	-	
Basic	9,026 (54.3)	176 (56.2)	19.5	-	-	-		
Secondary+	2,682 (16.1)	38 (12.1)	14.2	-	-	-		
Missing	382	7	-	-				
**Sex**								
Female	8,455 (49.7)	138 (43.1)	16.3	0.02	5,475 (48.9)	61 (43.6)	11.1	0.21
Male	8,561 (50.3)	182 (56.9)	21.3	5,732 (51.2)	79 (56.4)	13.8		
**First birth**								
No	12,132 (71.3)	191 (59.7)	15.7	<0.001	8,651 (77.3)	117 (83.6)	13.5	0.07
Yes	4,884 (28.7)	129 (40.3)	26.4	2,541 (22.7)	23 (16.4)	9.1		
Missing	-	-	15	0				
**Parity**								
1	4,884 (29.0)	129 (40.6)	26.4	<0.01	2,396 (21.9)	19 (14.4)	7.9	0.02
2	3,639 (21.6)	47 (14.8)	12.9	1,872 (17.1)	19 (14.4)	10.1		
3	3,114 (18.5)	54 (17.0)	17.3	1,721 (15.7)	25 (18.9)	14.5		
4+	5,185 (30.8)	88 (27.7)	17.0	4,975 (45.4)	69 (52.3)	13.9		
Missing	194	2	243	8				
**Multiple birth**								
Single	16,396 (96.4)	276 (86.3)	16.8	<0.001	10,658 (96.2)	126 (90.7)	11.8	0.001
Multiple	614 (3.6)	44 (13.8)	71.7	423 (3.8)	13 (9.4)	30.7		
Missing	6	0	126	1				
**Day of birth**								
Weekday	12,149 (71.4)	215 (67.2)	17.7	0.09	8,015 (71.5)	96 (68.6)	12.0	0.44
Weekend	4,867 (28.6)	105 (32.8)	21.6	3,192 (28.5)	44 (31.4)	13.8		
**ANC**								
Yes	-	-	-	8,142 (86.8)	103 (82.4)	12.7	0.15	
No	-	-	-	1,241 (13.2)	22 (17.6)	17.7		
Missing	-	-	-	1,824	15			

- Data not available.

### Neonatal mortality rate

**Figure 1.**
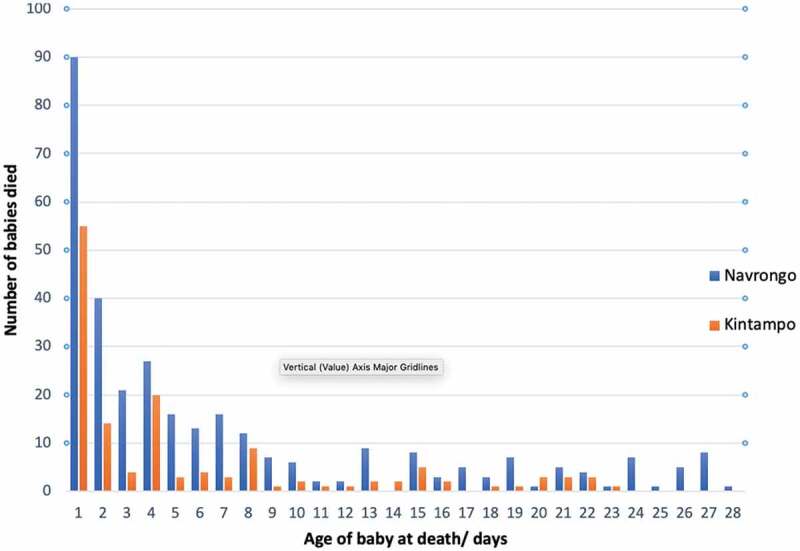
Distribution of neonatal deaths in Navrongo and Kintampo by age at death

Overall, 460 babies died within 28 days of birth, producing a combined NMR of 16.3. Of these, 326 (70.9%) died in the first week (0–6 days) (Figure 1). In Navrongo, 320 babies died within 28 days of birth (NMR 18.8): 223 (69.7%) within the first 6 days (early NMR 13.1) and 97 (30.3%) between 7 and 27 days (late NMR 5.7). Of the early neonatal deaths, 90 (40%) died during the first day. In Kintampo, 140 babies died within 28 days (NMR 12.5): 103 (73.6%) within the first 6 days (early NMR 9.2) and 37 (26.4%) between 7 and 27 days (late NMR 3.3). Of the deaths in the first week (0–6 days), 55 (53.4%) died during the first day. The NMR in Kintampo was one-third lower than in Navrongo, and the difference remained when the Navrongo data were restricted to 2005–2010 when birth information was available in both surveillance sites (p < 0.01).

The annual NMR decreased in Navrongo by 31% (from 18.6 in 2004 to 12.8 in 2012), and in Kintampo by 10.3%, from 12.6 in 2005 to 11.3 in 2010. A decline in NMR was observed for all birth locations but the rate of decline varied. In both Navrongo and Kintampo (Table 1), babies born in hospital had the highest NMR compared to clinic or home births. In Navrongo, clinic births had a lower NMR than home births (11.4 vs 19.9) but in Kintampo, they were similar (10.1 vs 10.0).

### Risk factors for neonatal mortality

In Navrongo, the factors associated with a higher overall neonatal mortality were hospital birth, male birth, multiple births, and year of birth, less maternal education and first birth (Table 2). In Kintampo they were: hospital birth, maternal age and multiple births and ANC non-attendance (Table 3). The adjusted population attributable fraction of overall neonatal deaths associated with non-attendance at ANC in the complete cases in Kintampo was 7% (95% CI: 3%-10%). The area under the ROC curve for the logistic regression model for complete cases was 68% each for Navrongo and Kintampo respectively, showing a moderate strength of the model. A Hosmer-Lameshow test to check the goodness of fit of the final model produced non-significant results at varying degrees of freedom, indicative of a good fit. When we combined the data for Navrongo and Kintampo, restricted to the years for which data were available in both regions, a bivariate analysis showed that hospital birth, male births, first births and multiple births were associated with an increased NMR. (p < 0.05).

**Table 2.** Multivariable logistic regression analyses of factors associated with neonatal mortality in Navrongo (2006–2012)

	Complete case model (N = 12,138)		Multiple imputations model (N = 13,327)	
	Multivariable OR (95% CI)*	p-value	Multivariable OR (95% CI)*	p-value
**Place of birth**				
Home	1.00	1.00		
Hospital	1.08 (1.02–1.14)	0.01	1.14 (1.03–1.25)	0.01
Clinic	0.67 (0.63–0.72)	<0.001	0.67 (0.65–0.68)	<0.001
**Maternal age**				
<20 years	1.18 (0.75–1.83)	0.48	1.14 (0.78–1.65)	0.50
20–29	1.00	1.00		
30–39	1.46 (1.04–2.05)	0.03	1.42 (0.98–2.06)	0.06
40+	0.72 (0.58–0.90)	<0.01	0.76 (0.64–0.91)	<0.01
**Socioeconomic status**				
Poor	1.47 (1.22–1.78)	<0.001	1.36 (0.97–1.90)	0.07
Next poor	1.33 (0.72–2.44)	0.36	1.25 (0.85–1.84)	0.27
Average	1.13 (0.77–1.68)	0.53	1.09 (0.65–1.81)	0.75
Next rich	1.20 (1.03–1.39)	0.02	1.14 (0.82–1.59)	0.42
Rich	1.00	1.00		
**Education**				
None	1.13 (1.06–1.21)	<0.001	1.12 (1.03–1.21)	0.01
Basic	1.00	1.00		
Secondary+	0.76 (0.69–0.84)	<0.001	0.76 (0.67–0.87)	<0.001
**Parity**				
1	1.00	1.00		
2	0.41 (0.21–0.78)	0.01	0.41 (0.23–0.74)	<0.01
3	0.54 (0.32–0.92)	0.02	0.58 (0.39–0.85)	0.01
4+	0.49 (0.31–0.77)	<0.01	0.52 (0.34–0.81)	<0.01
**Boys**	1.38 (1.18–1.61)	<0.001	1.39 (1.20–1.60)	<0.001
**Multiple birth**	5.06 (4.40–5.82)	<0.001	5.35 (5.16–5.55)	<0.001
**Weekend birth**	1.05 (0.79–1.39)	0.75	1.05 (0.75–1.45)	0.79
**Married**	1.03 (0.69–1.53)	0.89	1.17 (0.85–1.59)	0.33
**Year of birth**	0.98 (0.97–0.99)	<0.01	0.97 (0.97–0.98)	<0.001

*Adjusted for place of delivery, year of birth, maternal age, SES, marital status, education, sex, multiple birth, parity and day of birth.

*Data for multivariable analyses cover the years 2006–2012.

*m = 50.

**Table 3.** Multivariable logistic regression analyses of factors associated with neonatal mortality in Kintampo (2005–2010)

	Complete case model (N = 9,162)		Multiple imputations model (N = 11,207)	
	Multivariable OR (95% CI)#	p-value	Multivariable OR (95% CI)#	p-value
**Place of birth**				
Home	1.00	1.00		
Hospital	1.72 (1.57–1.88)	<0.001	1.76 (1.55–2.00)	<0.001
Clinic	1.27 (1.24–1.31)	<0.001	1.12 (1.06–1.18)	<0.001
**Maternal age**				
<20 years	1.07 (0.90–1.25)	0.42	0.95 (0.75–1.20)	0.65
20–29	1.00	1.00		
30–39	1.35 (1.00–1.82)	0.05	1.28 (1.02–1.62)	0.04
40+	2.64 (1.15–6.08)	0.02	2.62 (1.44–4.78)	<0.01
**Socioeconomic status**				
Poor	1.20 (0.91–1.59)	0.21	0.96 (0.82–1.14)	0.67
Next poor	0.94 (0.78–1.12)	0.48	0.76 (0.65–0.88)	<0.001
Average	0.99 (0.60–1.65)	0.98	0.75 (0.58–0.96)	0.03
Next rich	1.28 (0.86–1.91)	0.23	1.01 (0.79–1.29)	0.93
Rich	1.00	1.00		
**Parity**				
1	1.00	1.00		
2	1.62 (1.06–2.48)	0.03	1.17 (0.51–2.67)	0.72
3	1.99 (1.74–2.29)	<0.001	1.55 (0.92–2.63)	0.10
4+	1.31 (0.82–2.10)	0.26	1.13 (0.54–2.39)	0.74
**Boys**	1.33 (0.86–2.04)	0.20	1.24 (0.66–2.33)	0.51
**Multiple birth**	2.12 (0.66–6.77)	0.20	2.48 (1.08–5.66)	0.03
**Weekend birth**	0.93 (0.71–1.23)	0.63	1.14 (0.96–1.36)	0.14
**ANC attendance**	0.65 (0.50–0.84)	0.001	0.66 (0.49–0.90)	0.01
**Year of birth**	0.69 (0.48–0.81)	0.01	0.71(0.67–0.92)	0.04

#Adjusted for place of delivery, year of birth, maternal age, SES, sex, multiple birth, parity, antenatal care attendance and day of birth.

#Data for multivariable analyses cover the years 2005–2010.

#m = 40.

**Figure 2.**
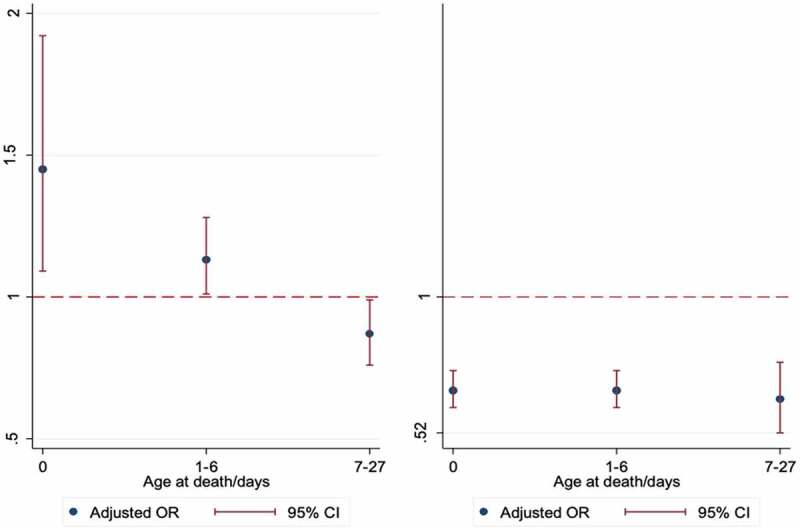
Forest plot of adjusted* odds ratio for the association between place of birth and neonatal mortality in Navrongo, according to age at death. (Left- Hospital births, Right- Clinic births, Reference line- Home births) Adjusted for place of delivery, year of birth, maternal age, SES, marital status, education, sex, multiple birth, parity, day of birth and year of birth

**Figure 3.**
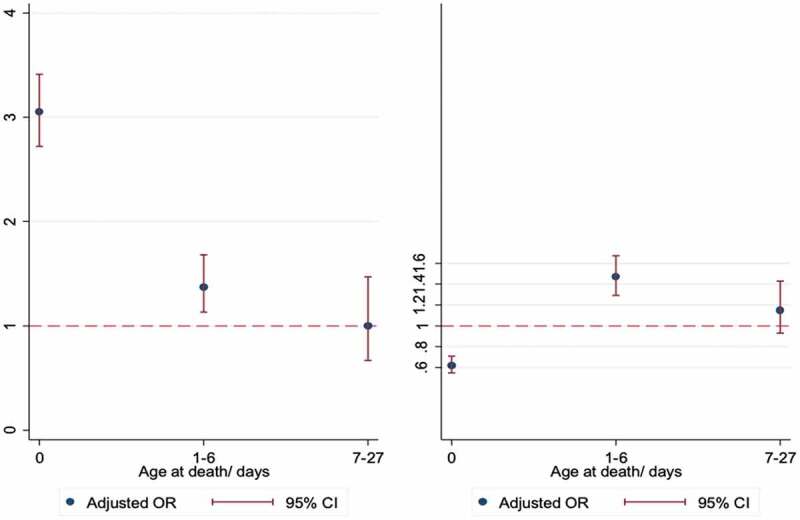
Forest plot of adjusted* odds ratio for the association between place of birth and neonatal mortality in Kintampo, according to age at death. (Left- Hospital births, Right- Clinic births, Reference line- Home births)

Subgroup analyses showed that in Navrongo, babies born in hospital were at a significantly increased risk of death on the day of birth and within days 1–6, but at a lower risk of death thereafter compared to babies born at home. Babies born in clinics remained at lower risk of death throughout the neonatal period, compared to home births (Figure 2). In Kintampo, hospital birth was a significant risk factor for early neonatal deaths- day 0 (adjusted OR 3.05, 95% CI: 2.72–3.41, p = 0.01) and days 1–6 (adjusted OR 1.37, 95% CI: 1.13–1.68, p < 0.01), but not late neonatal deaths (Figure 3). Compared to home births, clinic births were less likely to die on the day of birth (adjusted OR 0.62, 95% CI: 0.54–0.71, p < 0.01) but were significantly more likely to die between days 1–6 (adjusted OR 1.47, 95% CI: 1.29–1.67, p < 0.01).

### Place of death

**Figure 4.**
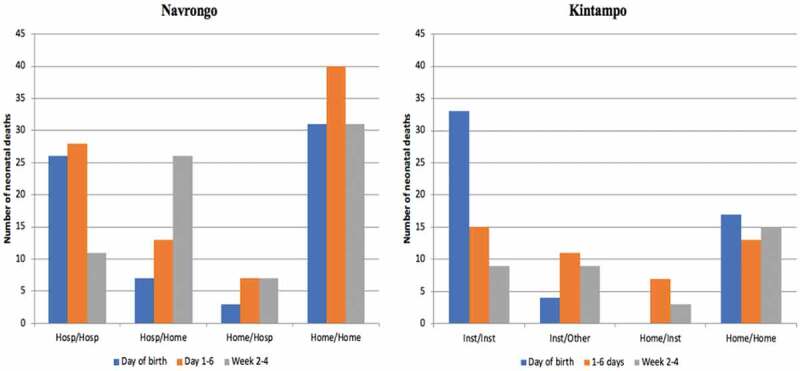
Number of neonatal deaths by place of birth/place of death according to age at death

Figure 4 shows the distribution of neonatal deaths based on where they were born and where they died (born/died). It shows that in Navrongo, more babies born at home and died at home (Home/Home) (n = 102, 82.3% of deaths of home births) compared to those born in hospital and died in hospital (Hosp/Hosp) (n = 65, 58.5% of deaths of hospital births). There were more deaths of hospital births at home (Hosp/Home) (n = 46, 41.4%), than deaths of home births in hospital (Home/Hosp) (n = 17, 13.7%). 15% of babies who were born in hospital but died at home (Hosp/Home) died on the day of birth, but most died after the first week. The distribution of neonatal deaths by place of birth and death remained similar when data for hospital and clinic deaths were combined (as health facility birth or death) compared to home births or deaths.

Likewise, in Kintampo, a higher proportion of babies born at home and also died at home (Home/Home) (n = 45, 81.8% of deaths of home births), compared to those born in a hospital or clinic (health facility birth) and also died in a hospital or clinic (health facility death) (n = 57, 70.4% of deaths of hospital births)- although the absolute numbers of those who were born in health facility and also died in the health facility is higher. There were more births in a health facility who died at home (n = 24, 29.6%) than home births who died in a health facility (n = 10, 18.2%). Among babies who were born in a health facility and also died in a health facility, most of them died on the day of birth, (n = 33, 89.2%) but among babies who were born at home and also died at home (Home/Home), the proportions of death were distributed more equally across the neonatal periods. Four babies (10.8%) who were born in a health facility died at home on the same day they were born, but none of the babies born at home died in a health facility on the day they were born (Figure 4).

### Causes of death

Overall, the leading causes of neonatal mortality were; infection (n = 121, NMR 4.3), asphyxia (n = 105, NMR 3.7) and prematurity/ low birth weight (n = 62, NMR 2.2). In Navrongo they were infection (29%, NMR 5.1), asphyxia (23%, NMR 4.2) and prematurity (18%, NMR 3.2)], and in Kintampo, infection (24%, NMR 3.0), asphyxia (24%, NMR 3.0), anaemia and other unspecified causes (21%, NMR 2.6), prematurity and low birth weight (5%, NMR 0.6). In both Navrongo and Kintampo, asphyxia and prematurity were the leading causes of death in the first six days, with asphyxia being the leading cause of death on the first day. Infection was the leading cause of death from 7–27 days. The differences in cause of death by age at death were statistically significant (p < 0.001) in both regions.

**Figure 5.**
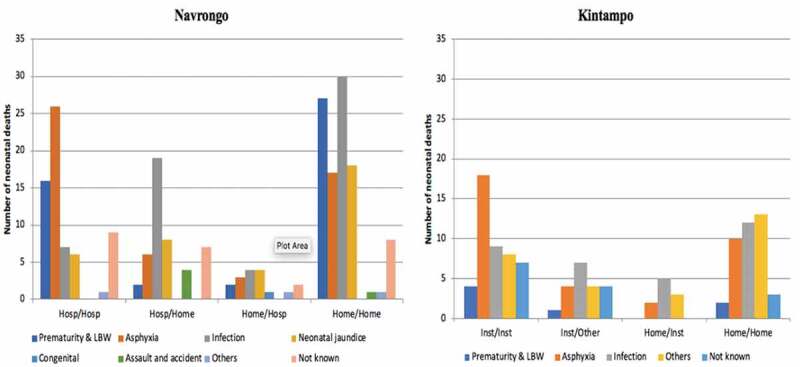
Cause of neonatal death by place of birth/place of death

The bar graph in Figure 5 shows that in Navrongo, the leading causes of death by place of birth/place of death were: Hosp/Hosp (asphyxia = 40%), Hosp/Home (infection = 42%), Home/Hosp (neonatal jaundice and infection = 24% each) and Home/Home (infection = 29%).

In Kintampo, the leading cause of death among babies who died in a health facility was asphyxia (n = 20, 35.7%) and the leading cause of death among babies who died at home was infection (n = 19, 31.7%). Among those who were born in a health facility but died at home (Facility/Home) and those who were born at home but died in a health facility (Home/Facility), the largest proportion died because of infection. The differences in cause of death by place of birth and death were not statistically significant.

## Discussion

### NMR

The overall NMRs in Kintampo and Navrongo were 12.5 and 18.8 respectively, with the annual rates declining in both areas during the study periods. These are lower than the national rate estimated in the 2014 Ghana Demographic and Health Survey (DHS, NMR = 29) [2] or the 2018 UN Inter-agency Group for Child Mortality Estimation (NMR = 24) [1]. Studies of NMR in Kintampo in 2003–4 and Navrongo in 2003–9 found NMRs of 31 and 24 respectively [22,23]. NMRs in the present study may thus not be representative of Ghana as a whole. This may reflect differences between the HDSS areas and other parts of the country. They could result from interventions and research initiatives which have occurred in these surveillance sites. For example, in Navrongo, the Community-based Health Planning and Services (CHPS) initiative assigned nurses to community locations to provide curative and preventive care including reproductive health care [24–26]. In Kintampo, the Newborn Health Intervention studies (NEWHINTS trials) also led to improved access to maternal care and significant reductions in maternal and infant mortality rates [27,28]. Further research is needed to explore the individual contributions of these interventions to the reduction in overall NMR, understand reasons which may explain the inequalities in NMR between HDSS sites and identify other programmes that could further reduce NMRs across the whole of Ghana, in order to achieve the third SDG of reducing NMRs to less than 12 by 2030.

### Place of delivery and neonatal mortality

Our findings corroborate results from the 2014 Demographic and Health Survey which reported that the majority of women in Ghana now deliver in a health facility. The Upper East region, where the Navrongo HDSS is situated, reported higher rates of facility deliveries than the Bono East region where the Kintampo HDSS is located [2]. The higher proportion of facility births in Navrongo may be attributed to the large number of CHPS compounds which have increased access to skilled delivery in the region [24,25]. It is expected that the proportion of facility deliveries will increase further following a recent directive by the Ministry of Health which bars TBAs from conducting deliveries and restricts their roles to referrals [29,30]. The lower proportion of facility births in Kintampo could be due to the slightly earlier period covered by the data (2005–2010) compared to Navrongo (2004–2012). It is hoped that future analyses based on more current data will reflect the increasing proportion of facility birth across the country [2].

Perceptions that increasing facility-based deliveries will automatically improve neonatal outcomes are overly simplistic and previous studies were inconclusive [6,31]. The present study found that hospital delivery had a higher overall NMR, and early neonatal mortality, than home deliveries in both areas. Clinic deliveries had the lowest NMR throughout the neonatal period in Navrongo, but in Kintampo were associated with a lower risk of death on the day of birth. These findings appear counterintuitive. However, the higher risk of death among hospital births could well be due to more high-risk pregnancies being delivered in hospitals, for example twin and first births, or high-risk pregnancy transfers from clinics and homes to hospitals [32–34].

An exploration of the neonatal death profiles revealed that the majority of deaths occurred at home, and of these, many had been born in hospital. There were more babies who were born in hospital but died at home, especially deaths which occurred on the first day and in the first week, compared to those born at home but died in hospital. In Navrongo, for example, 40% of the deaths of hospital births occurred at home, compared to only 14% who were born at home but died in hospital (Figure 2). In Kintampo, similar excess deaths were observed among hospital births at home. Also, a higher proportion of hospital births who died in hospital occurred in the first week where the risk of death is known to be high, but the proportion of home births who died at home was similar across the neonatal period.

It is unacceptable that babies born in a hospital go home to die on the same day of birth. This strongly suggests that babies born in hospital may have been discharged home too early without receiving appropriate immediate postnatal care or discharge assessment. The relatively small number of babies who are born at home but died in hospital may reflect the many challenges women and families face in seeking care for sick newborns, including such things as recognition of symptoms and the need for care, securing timely and affordable transportation, and obtaining high-quality, coordinated care once they arrive at the facility [35–37]. It is likely that recent widespread promotion of facility-based delivery in Ghana may have directly resulted in the decline in home deliveries [38]. As a consequence, the increased pressure on limited health facilities and insufficient human resources may have caused clinicians to discharge mothers too soon after delivery without proper assessment. Anecdotal evidence suggests that, in some hospitals, there are not enough beds to admit mothers and some sleep on the floor. Other contextual factors affecting care seeking for neonatal diseases, including cultural and belief patterns have also been noted [36–39]. Overemphasising where babies are born without considering where they are cared for, may therefore produce misleading results.

Improving postnatal care, particularly community-based care, for newborn illnesses by skilled personnel is an important policy recommendation from this study. This would involve equipping existing community nurses with the skills to assess sick babies at home, treating minor newborn ailments and referring more serious ones to a medically staffed service. They would require the support of an efficient referral system providing timely access to competent medical services. As this study also suggests, increasing the proportion of births in low quality hospitals will not reduce the NMR. A previous study in Ghana noted large gaps in ‘effective coverage’ of skilled birth attendance, defined as the proportion of births which occur in high quality clinical environments [40]. Ideally, this would include adequately resourcing hospitals to manage high-risk deliveries and keeping mother and baby a little longer in the health facilities to allow sufficient time for monitoring before discharge.

### Causes of death

Asphyxia and prematurity were the leading causes of death in the early neonatal period, with asphyxia responsible for the majority of deaths on the day of birth. Infection was the leading cause of late neonatal deaths. Overall, asphyxia was the leading cause of death among hospital births, whereas infection was the leading cause of home deaths and death in hospital after home birth. These are consistent with global, national and regional estimates and confirm that much of the burden of neonatal mortality is preventable [11,12]. In particular, they highlight the potential for reducing neonatal deaths due to asphyxia in health facilities and deaths due to infection at home.

The causes of asphyxia are well known, and the degree of injury caused by asphyxia is proportional to the duration of ischemia or hypoxia [41,42]. Fatal consequences of asphyxia are avoidable if appropriate interventions are taken on time: appropriate monitoring of mother and baby in utero before and during labour, resuscitation of the asphyxiated baby, and more advanced mechanical ventilation support [43]. The Ghana National Newborn Health Strategy and Action Plan [4] aimed to train at least 90% of skilled birth attendants in essential newborn care by 2018 but this does not appear to have been achieved. Such initiatives are encouraged and it is strongly recommended that efforts to meet this target are expedited by organising training workshops for all maternity care providers. These educational programmes should be complemented with ongoing skills practice, logistics, monitoring and refresher training or drills to ensure care providers maintain their skills.

That infection was the main cause of death among babies who were born and died at home could be related to poor conditions for home delivery, poor newborn care practices or delayed diagnosis and management. Previous studies have reported that practices such as the application of herbs to the umbilical cord could predispose babies to infection [44,45]. Common infections reported in central and northern Ghana include septicaemia, pneumonia, meningitis, diarrhoea and tetanus [22,23]. There is also evidence that the first point of call for mothers regarding neonatal illnesses is a relative, or traditional healer because of beliefs that newborn infections may be caused by spirits [37]. Hospitals often do not have essential laboratory apparatus to diagnose infections. Enabling medical services to accurately diagnose and treat neonatal infections, and improving access to postnatal care and public education to demystify spiritual causes of diseases are needed to encourage mothers to seek treatment from skilled care providers.

### Risk factors for neonatal mortality and place of death

Consistent with previous studies, this study found that babies born to educated mothers were less likely to die in the neonatal period compared to those born to uneducated mothers. However, it was interesting to note that the SES of the mother was not related to neonatal mortality. It has been suggested that widespread implementation of CHPS may have offset the dire consequences of poverty in northern Ghana [22]. In addition, the policy of free maternal delivery in Ghana ensures that all pregnant women can be registered with the National Health Insurance Scheme at no cost. This insurance also covers the newborn for the first three months. Thus, the need for direct healthcare expenditure by the mother has been reduced [46]. First births, twins and boys [22,34,47] were at significantly increased risk of neonatal mortality. Previous studies in the UK showed that twins, especially second twins delivered at term via vaginal delivery are at increased risk of death due to intrapartum anoxia [48,49]. It has been suggested that planned caesarean section could reduce deaths of twins by about 75% [48]. Boys may be at higher risk of death in the neonatal period due to lower birthweight [50] or a higher proportion of preterm deliveries [51]. Our results on parity and neonatal mortality were mixed. In Navrongo, first birth was associated with a higher risk of neonatal mortality as demonstrated in a previous meta-analysis and another multisite study [34,52], but we did not find this in Kintampo. Previous studies suggest that physiologic adaptation, prematurity and foetal growth may mediate the relationship between parity and mortality among neonates, but our study did not include these variables to allow formal analyses [53,54]. We did not find a conclusive association between maternal age and neonatal mortality.

## Strengths and limitations

This is one of the largest studies of causes of neonatal death in an African country. It used data from two geographically distinct and well-defined surveillance areas in Ghana, using robust and validated methods to collect data on both births and deaths. To our knowledge, it is the first to combine both place of birth and place of death into a single variable. By doing so, it provides a clearer understanding as to why babies born in hospital may be at higher risk of death. Future studies could focus more on neonatal deaths due to delivery related activities only, for example birth asphyxia.

The study has several limitations. First, the data for this study are rather dated (2005–2010 and 2004–2012) and so it is likely that there may have been changes in, for example, the proportion of births that occur in health facilities in the study settings. However, at the time of ethics application, more recent data had not been verified and were thus not available. Future studies based on more recent data will reveal trends in the proportion of births and should allow investigation of changes in mortality risk factors. In the two HDSS datasets analysed, there were relatively small numbers of missing data for some variables: we used multiple imputation techniques to model possible values for these. Some variables were recorded only for dead babies but not those who survived and so could not be included in the multivariable analyses. While neonatal deaths in this study could have been under-reported due to poor follow-up and recording, this is unlikely as frequent visits to each household are conducted to identify pregnancies, pregnancy outcomes and neonatal death. Finally, HDSS data do not typically include clinical data such as Apgar score, mode of delivery, presentation at birth, birth weight and maternal conditions/complications (preeclampsia and anemia), or other relevant societal and community level variables such as district level characteristics and the quality of health facilities. Some of these variables could influence neonatal outcomes and also potentially help explain high NMR in the study areas and differences between the two HDSS. It is recommended that the HDSSs consider revising their data collection tools to include these relevant variables, as resources allow.

## Conclusion

While the NMRs in the HDSSs appear lower than in Ghana as a whole, there is still potential for further reduction if deaths due to asphyxia and infections are reduced. Births in health facilities should be encouraged, by gradually phasing out home births, in conjunction with increased health service capacity. This should be complemented by enabling mothers to seek timely and appropriate help for neonatal illnesses and equipping health staff to manage these effectively. We believe that if such programmes that have already helped to reduce NMRs in the HDSSs are carefully rolled out nationwide, the third SDG of reducing NMRs to less than 12 by 2030 can be achieved in Ghana.

## Paper context

While almost all newborn deaths worldwide occur in developing countries, weak systems in these countries make studying such deaths and their causes difficult. Using data from well-established health surveillance systems in Ghana, this study showed that newborn deaths are reducing, but a significant proportion of babies still die of asphyxia in the first week, especially among those born in hospitals. Most neonatal deaths, however, occurred at home due to infections. Improving perinatal care and community-based postnatal care could substantially reduce newborn deaths.
